# Activation of μ‐opioid receptors by MT‐45 (1‐cyclohexyl‐4‐(1,2‐diphenylethyl)piperazine) and its fluorinated derivatives

**DOI:** 10.1111/bph.15064

**Published:** 2020-05-13

**Authors:** Daniel T. Baptista‐Hon, Mark Smith, Samuel Singleton, Lysbeth H. Antonides, Niamh Nic Daeid, Craig McKenzie, Tim G. Hales

**Affiliations:** ^1^ Institute of Academic Anaesthesia, Division of Systems Medicine, School of Medicine, Ninewells Hospital and Medical School University of Dundee Dundee UK; ^2^ Leverhulme Research Centre for Forensic Science, School of Science and Engineering University of Dundee Dundee UK

## Abstract

**Background and Purpose:**

A fluorinated derivative (2F‐MT‐45) of the synthetic μ‐opioid receptor agonist MT‐45 (1‐cyclohexyl‐4‐(1,2‐diphenylethyl)piperazine) was recently identified in a seized illicit tablet. While MT‐45 is a Class A drug, banned in a number of countries, nothing is known about the pharmacology of 2F‐MT‐45. This study compares the pharmacology of MT‐45, its fluorinated derivatives and two of its metabolites.

**Experimental Approach:**

We used a β‐arrestin2 recruitment assay in CHO cells stably expressing μ receptors to quantify the apparent potencies and efficacies of known (MT‐45, morphine, fentanyl and DAMGO) and potential agonists. In addition, the GloSensor protein was transiently expressed to quantify changes in cAMP levels. We measured Ca^2+^ to investigate whether MT‐45 and its metabolites have effects on GluN1/N2A NMDA receptors stably expressed in Ltk‐ cells.

**Key Results:**

The fluorinated MT‐45 derivatives have higher apparent potencies (2F‐MT‐45: 42 nM) than MT‐45 (1.3 μM) for inhibition of cAMP accumulation and β‐arrestin2 recruitment (2F‐MT‐45: 196 nM; MT‐45: 23.1 μM). While MT‐45 and 2F‐MT‐45 are poor recruiters of β‐arrestin2, they have similar efficacies for reducing cAMP levels as DAMGO. Two MT‐45 metabolites displayed negligible potencies as μ receptor agonists, but one, 1,2‐diphenylethylpiperazine, inhibited the NMDA receptor with an IC_50_ of 29 μM.

**Conclusion and Implications:**

Fluorinated derivatives of MT‐45 are potent μ receptor agonists and this may pose a danger to illicit opioid users. Inhibition of NMDA receptors by a metabolite of MT‐45 may contribute to the reported dissociative effects.

**Abbreviations**
DAMGO: ([d‐Ala^2^,NMe‐Phe^4^,Gly‐ol^5^]‐enkephalin)
M1: 1,2‐diphenylethylpiperazine
M17: 1‐cyclohexylpiperazine
MT‐45: 1‐cyclohexyl‐4‐(1,2‐diphenylethyl)piperazine
NMDA: *N*‐methy‐d‐aspartate
RLU: relative luminescence units

**What is already known**

- The synthetic opioids, MT‐45 and a fluorinated derivative, 2F‐MT‐45, are misused with potentially fatal consequences.

**What this study adds**

- Three fluorinated derivatives of MT‐45 potently activate μ receptors and one metabolite inhibits NMDA receptors.

**What is the clinical significance**

- The pharmacology of MT‐45, its derivatives and metabolites may contribute to the potential for harm.

## 1 INTRODUCTION

Up to 55% of people in developing countries and 19% in developed countries experience persistent pain (Noble et al., 2010). High levels of chronic pain contribute to increasing numbers of opioid prescriptions (Desai et al., 2019; Lee, Kremer, Guan, Greenberg, & Solomon, 2019; Torrance et al., 2018). Opioids remain among the most effective analgesics for treating moderate and severe acute pain, but their side effects (constipation, immune suppression and respiratory depression) are problematic (Colvin, Bull, & Hales, 2019; Williams et al., 2013). The development of analgesic tolerance and opioid‐induced hyperalgesia complicate the use of opioids in the treatment of chronic pain (Colvin et al., 2019). Furthermore, opioid exposure can be habit forming leading to addiction. Although prescription opioid‐related fatalities in the United States appear to have reached a plateau, illicit fentanyl and its analogues, including carfentanil, and other novel synthetic opioids (NSOs) are now contributing to a new wave of deaths (Socías & Wood, 2017). The addictive potential and profitability of opioids has driven a proliferation of novel synthetic opioids (Prekupec, Mansky, & Baumann, 2017).

MT‐45 (1‐cyclohexyl‐4‐(1,2‐diphenylethyl)piperazine) is an novel synthetic opioid originally developed as a potential analgesic substitute for morphine (Fujimura, Tsurumi, Nozaki, Hori, & Imai, 1978; Nakamura & Shimizu, 1976). Research into MT‐45 was discontinued shortly thereafter due to its side effect profile. However, a recent study demonstrated that MT‐45 has a higher affinity for μ receptors than for either δ‐ or κ‐receptors (Baumann et al., 2018). It is now a Class A restricted substance in the United Kingdom and banned in a number of other countries. A fluorinated derivative of MT‐45 (2F‐MT‐45) was discovered in a tablet seized at a music festival in 2016. The syntheses of MT‐45 and 2F‐MT‐45 were recently described (McKenzie et al., 2018). Analysis of MT‐45's in vitro and in vivo metabolism led to the identification of two major metabolites, 1,2‐diphenylethylpiperazine (M1) and 1‐cyclohexylpiperazine (M17).

Opioids initiate their analgesic and adverse effects by activating μ‐opioid receptors (Matthes et al., 1996). Activated μ receptors signal via G_i/o_‐proteins to inhibit adenylate cyclase activity, causing a reduction in cAMP levels. Activation of G_i/o_‐proteins also increases K^+^ conductance, via activation of G protein activated inwardly rectifying K^+^ channels and inhibits voltage‐activated Ca^2+^ channel activity (Williams et al., 2013). The resulting reduction in neuronal excitability, particularly in the pain pathways, underlies the analgesic effects of opioids. However, activation of μ receptors outside the pain pathways contributes to the detrimental effects of opioids, including respiratory depression and addiction, which are key contributors to rising drug death statistics in the United States and United Kingdom (Dwyer‐Lindgren et al., 2018; Kimber, Hickman, Strang, Thomas, & Hutchinson, 2019; Middleton, McGrail, & Stringer, 2016).

In addition to initiating G protein‐mediated signalling, activated μ receptors also recruit the multifunctional scaffold protein, β‐arrestin2 (Latorraca et al., 2018; Williams et al., 2013). β‐arrestin2 participates in μ receptor internalisation and recycling, as well as in the recruitment of additional kinases. Recruitment of β‐arrestin2 has been implicated in the detrimental effects of μ receptor agonists (Bohn et al., 1999; Raehal & Bohn, 2011). There is considerable interest in the idea that μ receptor agonists may be more or less prone to tolerance, respiratory depression and constipation when biased in favour or against β‐arrestin2 recruitment, respectively (Ehrlich et al., 2019).

User experiences of MT‐45 posted within online drug forums include analgesia, euphoria, tolerance, respiratory depression and constipation, consistent with other μ receptor agonists, as well as dissociative‐like effects akin to those reported by users of ketamine (Kjellgren, Jacobsson, & Soussan, 2016). The cause of the apparent dissociative effects of MT‐45 is unclear. It is not known whether MT‐45 or its metabolites inhibit the activity of the NMDA receptor, the target of several dissociative drugs, including ketamine. The identification of metabolites of MT‐45 reveals that some have structural similarities to another dissociative, NMDA receptor‐inhibiting drug, diphenidine (McKenzie et al., 2018).

In this study, we characterised the pharmacology of MT‐45, 2F‐MT‐45 and its regioisomers, 3F‐MT‐45 and 4F‐MT‐45, along with two of the major metabolites of MT‐45, M1 and M17. We compared their potencies and efficacies with those of morphine, fentanyl and the synthetic peptide agonist DAMGO. We also examined the ability of MT‐45 and its metabolites, M1 and M17, to inhibit NMDA receptors.

## 2 METHODS

### 2.1 Cell culture and transfections

PathHunter CHO cells (DiscoverX, UK; RRID:CVCL_KY70) stably expressing β‐galactosidase fragment tagged human μ receptors and β‐arrestin2 were maintained in DMEM supplemented with F12 nutrient mixture (1:1 ratio with DMEM), 10% FBS, penicillin (5,000 units·ml^−1^) and streptomycin (5,000 μg·ml^−1^). Mouse fibroblast Ltk‐ cells, stably expressing the inducible NMDA receptor NR1A and NR2A subunits (McIlhinney et al., 1998), were maintained in DMEM supplemented with 10% FBS, penicillin (5,000 units·ml^−1^) and streptomycin (5,000 μg·ml^−1^). Stable expression of tagged μ receptors and β‐arrestin2 in PathHunter CHO cells was maintained by the presence of geneticin (500 μg·ml^−1^) and hygromycin B (250 μg·ml^−1^). Stable expression of NMDA receptor GluN1 and GluN2A subunits in Ltk‐ cells was maintained by the presence of geneticin (500 μg·ml^−1^). All cells were cultured at 37°C in a humidified atmosphere in 95% air.

For transfections, 1 × 10^6^ CHO cells, plated onto 35 mm dishes, were left to settle overnight. The pGloSensor (Promega, UK) cDNA (2 μg) was transfected into PathHunter CHO cells using Lipofectamine (Invitrogen, UK) according to the manufacturer's instructions. Transfection success was verified by performing parallel GFP cDNA transfections (2 μg) under the same conditions. GFP was visualised using fluorescence microscopy.

### 2.2 GloSensor cAMP accumulation assay

CHO cells transfected with pGloSensor were suspended in OptiMEM (Invitrogen, UK), seeded onto 96‐half well plates at a density of 1.5 × 10^4^ cells per well and left to settle overnight. The medium was subsequently replaced with assay buffer containing: HBSS supplemented with 20 mM HEPES, 3 mM luciferin, 30 μM forskolin at pH 7.4. Cells were left to incubate in assay buffer for 2 h at room temperature. Luminescence as a result of forskolin‐stimulated accumulation of cAMP (maximum luminescence) was recorded on a GloMAX Navigator (Promega, UK) at 1 s integration. Agonists were diluted into assay buffer and added to the cells and incubated for 30 min at room temperature. Luminescence was read again at the end of the 30 min incubation with agonists.

### 2.3 β‐arrestin2 recruitment assay

The PathHunter β‐arrestin2 assay was used to assess recruitment of β‐arrestin2 to μ receptors. CHO cells, stably expressing β‐galactosidase fragment tagged human μ receptors and β‐arrestin2, were suspended in OptiMEM (Invitrogen, UK), seeded onto 96‐half well plates (Greiner, UK) at a density of 5 × 10^3^ cells per well and left to settle overnight. Agonists were diluted into OptiMEM. Cells were incubated in the presence of agonists for 90 min at 37°C. The substrate reagent for luminescence production (DiscoverX, UK) was added according to manufacturer's instructions and incubated for 2 h at 37°C before the luminescence was recorded.

### 2.4 Intracellular Ca^2+^ imaging and Flexstation measurements

Ltk‐ cells stably expressing inducible NMDA receptor GluN1A and NR2A subunits were seeded at a density of 5 × 10^4^ cells onto 16 mm glass coverslips or into each well of a black 96‐well plate. Cells were incubated overnight in growth media supplemented with 1 μM dexamethasone (to induce expression of NMDA receptor subunits) and up to 200 μM AP‐5 (to block activation of NMDA receptors by glycine and glutamate contained in growth media). The recording buffer composed of nominally Mg^2+^ free HBSS, supplemented with 2 mM CaCl_2_, 20 mM HEPES and 2 mM probenecid. The pH was adjusted to 7.4 with NaOH. Cells were loaded with 2 μM Fura2‐AM at room temperature for 1 h, followed by de‐esterification in recording buffer for at least 30 min. All buffers during the loading and de‐esterification steps contain 200 μM AP‐5. Coverslips were mounted in a chamber (RC‐25, Warner Instruments) and constantly superfused with recording buffer at a rate of approximately 5 ml·min^−1^ using gravity feed. Intracellular Ca^2+^ in single cells was measured using an inverted epifluorescence microscope with a 40× oil immersion objective. Fura‐2 was excited at 340 or 380 nm, selected using a monochromator (Cairn, UK). Emission at 510 nm was selected using a dichroic filter (Chroma Tech. Corp., USA) and collected using a cooled intensified photometric camera (Photometrics CoolSNAP HQ, Roper Scientific). Fluorescence and images were acquired using MetaFluor software version 5.0r3 (Universal Imaging Corp., UK; RRID:SCR_014294). For Flexstation Ca^2+^ measurements, agonists (NMDA and glycine) were added using the automated addition mode of the instrument from 10× drug stocks. Fluorescence excited at 340 or 380 nm was collected at 510 nm every 5 s. A baseline of 2 min was recorded before the addition of agonists.

### 2.5 Materials

**FIGURE 1:**
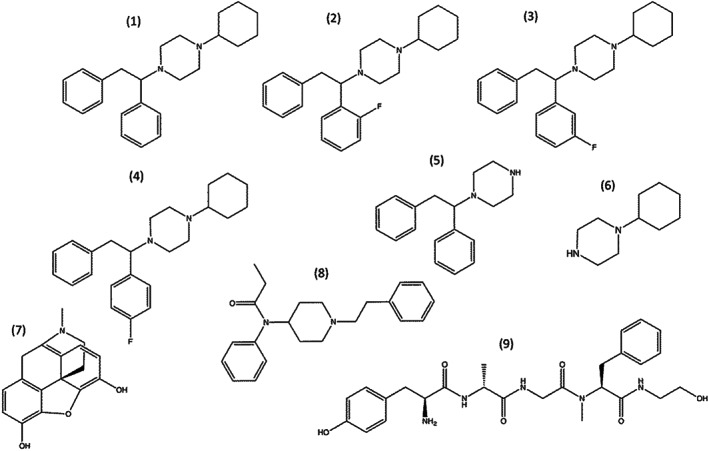
Chemical structures of tested compounds. (1) MT‐45, (2) 2F‐MT‐45, (3) 3F‐MT‐45, (4) 4F‐MT‐45, (5) MT‐45 metabolite M1, (6) MT‐45 metabolite M17, (7) Morphine, (8) Fentanyl and (9) DAMGO

All chemical structures used in this study are depicted in Figure 1. Fentanyl citrate, morphine sulphate and DAMGO ([d‐Ala^2^,NMe‐Phe^4^,Gly‐ol^5^]‐enkephalin) were purchased from Sigma‐Aldrich, UK. Luciferin was purchased from Promega, UK. AP‐5 and forskolin was purchased from HelloBio, UK. The syntheses of MT‐45, 2F‐MT‐45, 3F‐MT‐45 and 4F‐MT‐45 have been described previously (McKenzie et al., 2018). 1‐Cyclohexylpiperazine (M17) was purchased from Fluorochem, UK. All other chemicals were from Sigma‐Aldrich, UK.

### 2.6 Synthesis of the M1 metabolite of MT‐45

The hydrochloride salt of 1,2‐diphenylethylpiperazine (M1) was prepared using an adaption of the method reported by Geyer et al. (2016). Zinc dust (2.0 g, 30 mmol) was suspended in acetonitrile (40 ml) in a dried round‐bottom flask, which was flushed with argon to create an inert atmosphere. To this mixture was added benzyl bromide (0.4 ml, 3.3 mmol) and trifluoracetic acid (0.2 ml) and stirred for 5 min. Subsequently, benzyl bromide (3 ml, 25 mmol), 1‐Boc‐piperazine (1.87 g, 10 mmol) suspended in acetonitrile and benzaldehyde (1.2 ml, 11 mmol) were introduced to the solution. This mixture was stirred for an additional hour at ambient temperature. The resulting solution was quenched with a saturated aqueous ammonium chloride solution (150 ml) and extracted with dichloromethane (2 × 100 ml). The organic layers were dried with magnesium sulphate and concentrated in vacuo. The resulting yellow oil was dissolved in diethyl ether (150 ml) and treated with sulfuric acid (0.75 ml) to remove the Boc‐protective group. The mixture was stirred for 1 h, after which the ether was decanted off. The residue was re‐dissolved in an aqueous sodium hydroxide solution (1.25 M, 150 ml). The solution was extracted with dichloromethane (2 × 100 ml) and the organic layers were combined, dried with magnesium sulphate and concentrated *in vacuo.* The resulting yellow oil was purified using gravity column chromatography, with a mobile phase of dichloromethane‐methanol (90:10 v/v) and 0.8% ammonia. The resulting oil was dissolved in diethyl ether (200 ml) and treated with hydrogen chloride (4 M in dioxane, 6 ml, 24 mmol). This solution was left to stand for 5 min. The crystallised product was filtered and collected.

The synthesised product (yield 9.6%, purity 99.1%) was characterised using NMR and gas chromatography coupled to MS (GC–MS). ^1^H‐NMR (10 mg·ml^−1^ in D_2_O) and ^13^C‐NMR (20 mg·ml^−1^ in D_2_O) spectra were acquired on a JEOL ECS‐400 NMR spectrometer (JEOL, Tokyo, Japan), operating at 400 MHz. GC–MS analysis was performed on an Agilent Technologies 7890B GC system with 5977B MS Detector and 7,693 autosampler (Agilent Technologies, Wokingham, UK), using a capillary column (HP5 MS, 30 m, Å ~ 0.25 mm, i.d. 0.25 μm). Helium was used as the carrier gas at a constant flow rate of 1.0 ml·min^−1^. Injection temperature was 265°C and the detection temperature was 300°C. The temperature program was 60‐15‐300 (2′) with a split ratio of 50:1. Eicosane was used as an internal standard (t_R_ = 11.79).

### 2.7 M1 characterisation

^1^H‐NMR (400 MHz, D_2_O) δ 7.25–7.31 (m, 5H), 7.00–7.06 (m, 3H), 6.97 (dd, *J* = 7.1, 5.3 Hz, 2H), 4.59–4.63 (m, 1H), 3.29–3.56 (m, 10H); ^13^C‐NMR (101 MHz, D_2_O) δ 135.00 (s, 1C), 130.58 (s, 1C), 129.98 (s, 2C), 129.84 (s, 1C), 129.40 (s, 4C), 128.68 (s, 2C), 127.14 (s, 1C), 72.30 (s, 1C), 46.74 (s, 2C), 40.88 (s, 2C), 34.95 (s, 1C); GC–MS tR = 13.08 min, *m/z* = 266 [M^+^], 175 (100%), 91 (33%), 179 (15%), 176.1 (15%), 180 (13%).

### 2.8 Data analysis

Luminescence measurements, resulting from cAMP accumulation following the application of agonists, were expressed as a percentage of forskolin‐stimulated maximum luminescence. Luminescence data from the β‐arrestin2 assay were normalised to the maximum luminescence in the presence of DAMGO. Concentration–response relationships were plotted and fitted using a logistics equation:

$$f\left(\left[\text{Agonist}\right]\right)=\frac{\left(\mathrm{Max}-\mathrm{Min}\right)}{1+10^{\left(\log{\mathrm{EC}}_{50}-\left[\text{Agonist}\right]\times n^{H}\right)}},$$

where apparent potency (EC_50_), Hill slope (n^H^) and efficacy (E_max_: Max − Min) parameters were derived.

Fura‐2 emission fluorescence values collected from excitation at either 340 or 380 nm were expressed as a ratio (340 nm/380 nm). Amplitudes were calculated from background subtracted peak ratio fluorescence induced by glutamate and glycine application.

### 2.9 Statistics

The data and statistical analysis comply with the recommendations of the *British Journal of Pharmacology* on experimental design and analysis in pharmacology (Curtis et al., 2018). Summary data are presented as box and whisker plots, where the boundary of the boxes represent the interquartile range and the error bars represent the 5th to 95th percentile. Data median divides the boxes and data means are indicated as “+.” All box plots are visually inspected for normality and skewedness. In‐text summary data and concentration–response relationship data are presented as mean ± SEM, except EC_50_ and IC_50_ data, where mean and ranges are reported. Sample sizes represent independent experiments and statistical comparisons were performed using data on these independent values, and also where the sample size was at least 5. Pairwise comparisons (for intracellular Ca^2+^ data) were performed using the Student's paired *t*‐test. Comparisons of parameters derived from the logistics fit (pEC_50_ or pIC_50_, n^H^ and E_max_) between different agonists were performed using one‐way ANOVA. Post‐hoc testing was conducted using the Bonferroni correction method if *F* in the one‐way ANOVA had *P* < 0.05 and there was no significant variance inhomogeneity. Differences were considered statistically significant when *P* < 0.05.

### 2.10 Nomenclature of targets and ligands

Key protein targets and ligands in this article are hyperlinked to corresponding entries in http://www.guidetopharmacology.org, the common portal for data from the IUPHAR/BPS Guide to PHARMACOLOGY (Harding et al., 2018), and are permanently archived in the Concise Guide to PHARMACOLOGY 2019/20 (Alexander et al., 2019, 2019).

## 3 RESULTS

### 3.1 MT‐45 and its fluorinated derivatives are poor recruiters of β‐arrestin2

We used the PathHunter β‐arrestin2 recruitment assay to determine the potency and efficacy of MT‐45, the fluorinated derivative identified within a seized tablet, 2F‐MT‐45, and two other reference fluorinated derivatives (3F‐MT‐45 and 4F‐MT‐45). These were compared to the equivalent values obtained for established μ receptor agonists: DAMGO, morphine and fentanyl. All chemical structures are depicted in Figure 1.

**FIGURE 2:**
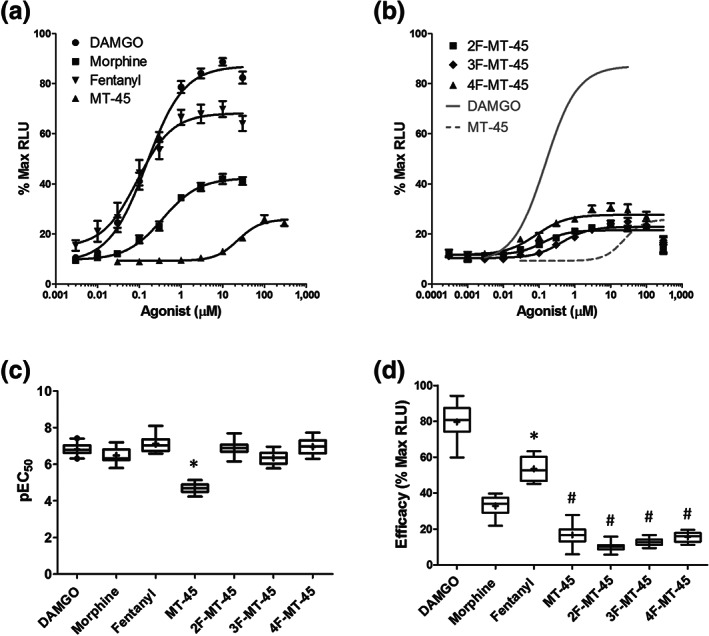
Recruitment of β‐arrestin2 to the μ receptor. (a) Concentration–response relationship of DAMGO, morphine, fentanyl and MT‐45 and (b) the fluorinated derivatives for recruitment of β‐arrestin2. Data are normalised to maximum recruitment. A logistics function was fitted to the data which yielded EC_50_, Hill slope, and efficacy parameters. The logistics fit to DAMGO and MT‐45 data are reproduced in (b) (grey lines) for comparison. (c) Box plots of pEC_50_ values from the data in (a) and (b). The details of the box plots are described in Section 2. A one‐way ANOVA was used to compare mean pEC_50_ (*F*_6, 82_ = 84.5; *P* < 0.05). A post hoc pairwise comparison with the Bonferroni correction revealed a statistically significant difference in the pEC_50_ of MT‐45 when compared to all the other agonists (*P < 0.05). (d) Box plots of efficacy values from the data in (a) and (b). A one‐way ANOVA revealed statistically significant differences in mean efficacies (*F*_6, 82_ = 251; *P* < 0.05). A post hoc pairwise comparison with the Bonferroni correction revealed fentanyl to be significantly different to all the other agonists (*P < 0.05). The analysis also revealed that MT‐45 and all its fluorinated derivatives are significantly less potent than DAMGO (^#^ *P* < 0.05)

PathHunter CHO cells were seeded onto 96‐half well plates and exposed to different concentrations of the agonists. Following agonist incubation, the cells were lysed and μ receptor‐β‐arrestin2 complexes were quantified using the manufacturer's luminescence detection solution. Luminescence was read on a plate reader and the relative luminescence values (RLU) were normalised to the maximum on the same plate, which always occurred at one of the saturating concentrations of DAMGO. For clarity, the concentration–response relationships of DAMGO, morphine, fentanyl and MT‐45 are plotted in Figure 2a, while those of the fluorinated derivatives are plotted in Figure 2b; the latter, together with the logistic fits of DAMGO (grey solid line) and MT‐45 (grey dotted line) are plotted for comparison.

Box plots for pEC_50_ values are shown in Figure 2c. The mean and range for the EC_50_ values are shown in Table 1. A one‐way ANOVA of the pEC_50_ values reveals a statistically significant difference between the data. We subsequently performed pair‐wise comparisons between all of the pEC_50_ values with the Bonferroni correction. The analysis reveals that MT‐45 is significantly less potent than all of the other agonists tested.

**TABLE 1:** Logistics fit parameters of concentration–response data for β‐arrestin 2 recruitment assay. RLU ‐ relative luminescence units

Agonist (*n*)	EC_50_ (nM)	Efficacy (% max RLU)	Hill slope
DAMGO (21)	200 (450)	78 ± 3.5	0.97 ± 0.11
Morphine (15)	470 (1,600)	33 ± 2.0	1.0 ± 0.16
Fentanyl (8)	120 (260)	54 ± 6.0	1.0 ± 0.29
MT‐45 (18)	23,000 (50,000)	17 ± 1.1	1.6 ± 0.33
2F‐MT‐45 (9)	200 (680)	10 ± 0.51	1.3 ± 0.51
3F‐MT‐45 (9)	610 (1,600)	12 ± 1.1	1.1 ± 0.33
4F‐MT‐45 (9)	170 (490)	16 ± 2.0	1.0 ± 0.37

*Note.* EC_50_ data are presented as mean (range). Other data are presented as mean ± SEM.

We measured the efficacy for β‐arrestin2 recruitment as the difference between the top and bottom of the logistic function fit. Box plots for efficacies are shown in Figure 2d, while the means (±SEM) are summarised in Table 1. A one‐way ANOVA reveals a statistically significant difference in mean efficacies. Pair‐wise comparisons between all mean efficacy values, with the Bonferroni correction reveal DAMGO to be significantly more efficacious than all of the other agonists . This is consistent with our previous finding that morphine is a partial agonist in terms of β‐arrestin2 recruitment (Bull et al., 2017). Our data also suggest that fentanyl is a partial agonist in this assay. The analysis reveals that MT‐45 and all of its fluorinated derivatives are significantly less efficacious than DAMGO, morphine and fentanyl.

**FIGURE 3:**
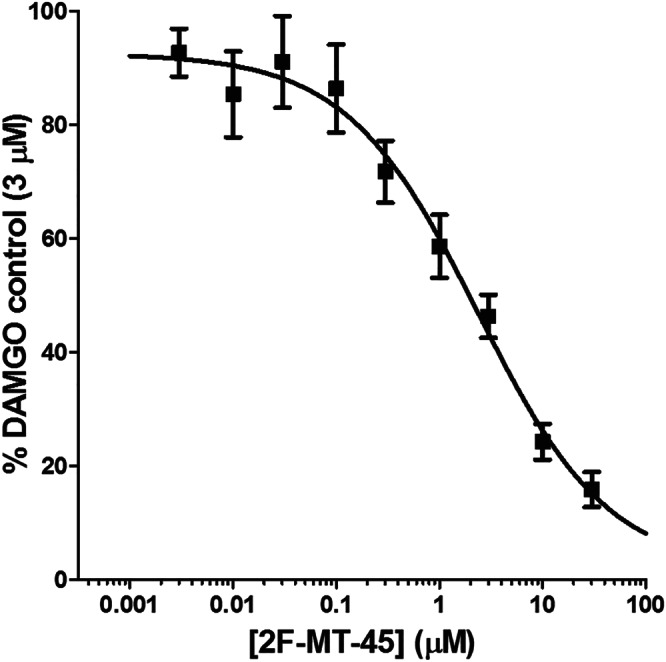
Inhibition of DAMGO‐mediated β‐arrestin2 recruitment by 2F‐MT‐45. Concentration–response relationship of 2F‐MT‐45 inhibition of DAMGO (3 μM) stimulated β‐arrestin2 recruitment. Data are normalised to maximum recruitment in the presence of DAMGO (3 μM). The fitted line represents the logistics function fit to the data

The fluorinated derivatives, including the newly discovered 2F‐MT‐45, are similar in terms of their low efficacies to recruit β‐arrestin2 as the parent compound, MT‐45. We therefore investigated whether 2F‐MT‐45 antagonises the effect of DAMGO, as would be anticipated for a partial agonist. Increasing concentrations of 2F‐MT‐45 (up to 30 μM) were applied with a maximally efficacious concentration of DAMGO (3 μM) to PathHunter CHO cells. Relative luminescence units was normalised to that of DAMGO (3 μM) alone and plotted as a concentration–response relationship (Figure 3). A logistics function was fitted to the data yielding a mean IC_50_ value (and range) for 2F‐MT‐45 of 2.3 (2.1) μM.

#### 3.2 MT‐45 and its fluorinated derivatives show differential apparent potencies as inhibitors of cAMP.

We used the GloSensor protein to assess μ receptor‐mediated changes in intracellular cAMP levels utilising the PathHunter CHO cells. This provides consistency of μ receptor density when making comparisons of apparent potency and efficacy between the two assays.

**FIGURE 4:**
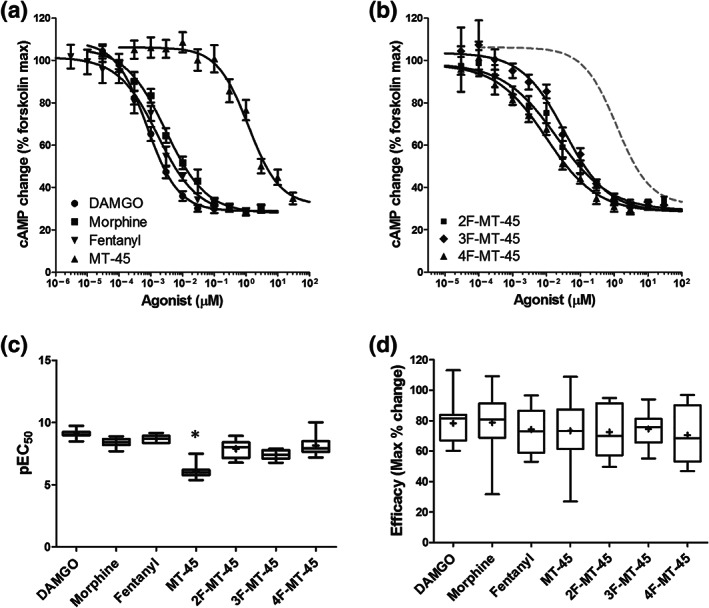
cAMP inhibition by μ receptor activation. (a) Concentration–response relationship of DAMGO, morphine, fentanyl and MT‐45 and (b) the fluorinated derivatives for reduction of cAMP levels in PathHunter CHO cells transiently expressing the GloSensor protein. Data are normalised to the maximum cAMP levels in the presence of forskolin (30 μM). A logistics function was fitted to the data which yielded IC_50_, Hill slope and efficacy parameters. The logistics fits to DAMGO and MT‐45 are reproduced in (b) (grey lines) for comparison. (c) Box plots of pIC_50_ values from the data in (a) and (b). The construction of the box plots is described in Section 2. A one‐way ANOVA was used to compare mean pIC_50_ (*F*_6, 67_ = 53.1; *P* < 0.05). A post hoc pairwise comparison with the Bonferroni correction revealed MT‐45 to be significantly less potent than all of the other agonists (*P < 0.05). (d) Box plots of efficacy values from the data in (a) and (b). A one‐way ANOVA revealed no statistically significant differences in mean efficacies (*F*_6, 67_ = 0.31; *P* = 0.93)

PathHunter CHO cells transfected with the GloSensor protein were plated onto 96‐half well plates and incubated in assay buffer containing forskolin (30 μM) to stimulate AC activity. Peak luminescence levels were subsequently measured, before addition of agonists. Agonist‐mediated reduction in luminescence was assessed after 30 min exposure. The luminescence values following agonist exposure were normalised to those prior to agonist addition and plotted against agonist concentrations. Similar to the β‐arrestin2 assay data above, the concentration–response relationships for DAMGO, morphine, fentanyl and MT‐45 are plotted in Figure 4a, while those for 2F‐, 3F‐, and 4F‐MT‐45 are plotted in Figure 4b, together with the logistics fits for DAMGO (grey solid line) and MT‐45 (grey dotted line) for comparison.

Box plots for pEC_50_ values are shown in Figure 4c. The mean and range for the EC_50_ values are shown in Table 2. A one‐way ANOVA analysis reveals a statistically significant difference in mean pEC_50_ values. We subsequently performed pair‐wise comparisons between all of the pEC_50_ values with the Bonferroni correction. Our analysis reveals that MT‐45 is significantly less potent than all of the compounds tested. Similar to above, this analysis reveals that all of the fluorinated derivatives are significantly more potent than MT‐45. The potencies of the three fluorinated derivatives were similar.

**TABLE 2:** Logistics fit parameters of concentration–response data for cAMP accumulation assay

Agonist (*n*)	EC_50_ (nM)	Efficacy (% forskolin max)	Hill slope
DAMGO (13)	1.0 (3.3)	80 ± 4.5	0.87 ± 0.11
Morphine (10)	5.4 (19)	78 ± 6.0	0.68 ± 0.16
Fentanyl (9)	2.5 (3.8)	74 ± 6.7	0.70 ± 0.15
MT‐45 (17)	1,300 (4,300)	74 ± 8.2	0.88 ± 0.15
2F‐MT‐45 (9)	42 (170)	69 ± 7.8	0.55 ± 0.12
3F‐MT‐45 (9)	58 (160)	75 ± 5.0	0.68 ± 0.11
4F‐MT‐45 (9)	18 (68)	70 ± 6.2	0.58 ± 0.21

*Note.* EC_50_ data are presented as mean (range). Other data are presented as mean ± SEM.

We measured the efficacy for cAMP reduction as the difference between the top and bottom of the logistic function fit. Box plots for efficacies are shown in Figure 4d, while the means (±SEM) are shown in Table 2. In contrast to β‐arrestin2 recruitment data, mean efficacies of all the agonists for cAMP reduction did not differ from each other.

#### 3.3 MT‐45 metabolites show negligible agonist activity at μ receptors, but M1 antagonises the NMDA receptor

**FIGURE 5:**
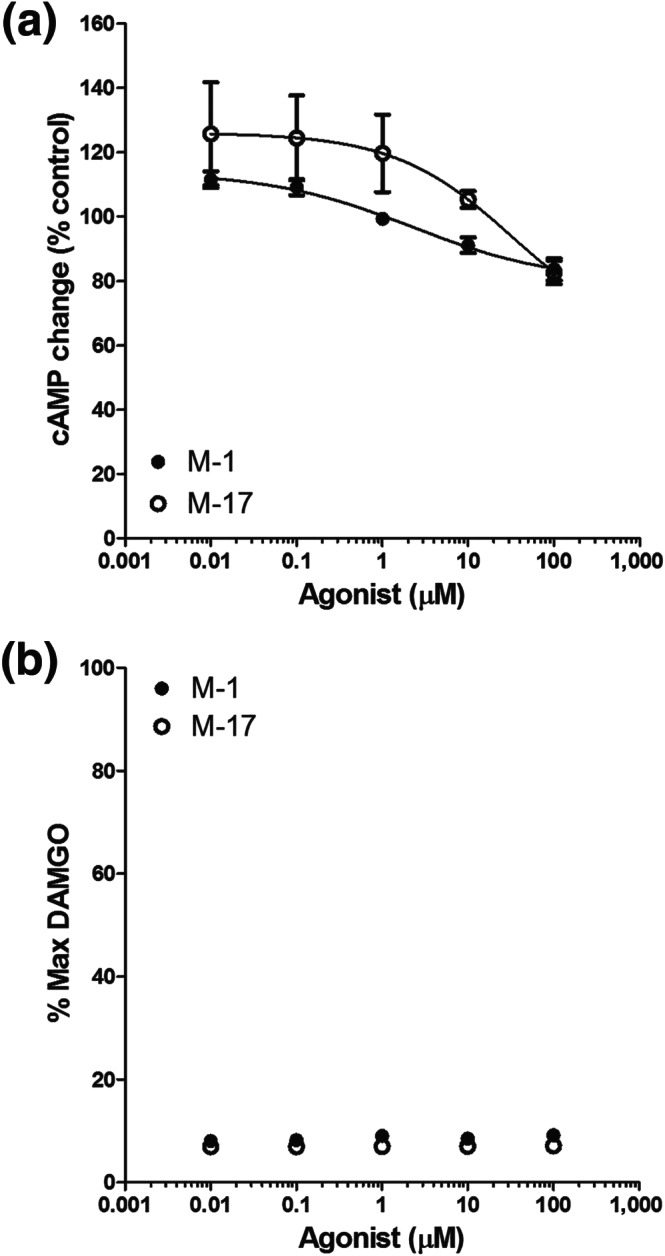
Effect of MT‐45 metabolites M1 and M17 on μ receptor function. (a) Concentration–response relationship of M1 and M17 on cAMP inhibition mediated by μ receptor activation. Both compounds produced very modest inhibition of cAMP levels at concentrations up to 100 μM. The solid lines indicate the logistic function fit to the data points. (b) Concentration–response relationship of M1 and M17 on β‐arrestin2 recruitment to μ receptors. Both compounds did not cause any β‐arrestin2 recruitment at concentrations up to 100 μM

Metabolites of MT‐45 have been identified using in vitro and in vivo data (McKenzie et al., 2018). It is not known whether these molecules possess any μ receptor agonist efficacy. We investigated the abilities of two major metabolites, M1 and M17, to activate μ receptors using the GloSensor cAMP assay and the PathHunter β‐arrestin2 recruitment assay. We found that both M1 and M17 compounds only showed very modest efficacy in reducing cAMP accumulation at concentrations up to 100 μM (Figure 5a). Neither compound showed any efficacy in the β‐arrestin2 recruitment assay (Figure 5b).

**FIGURE 6:**
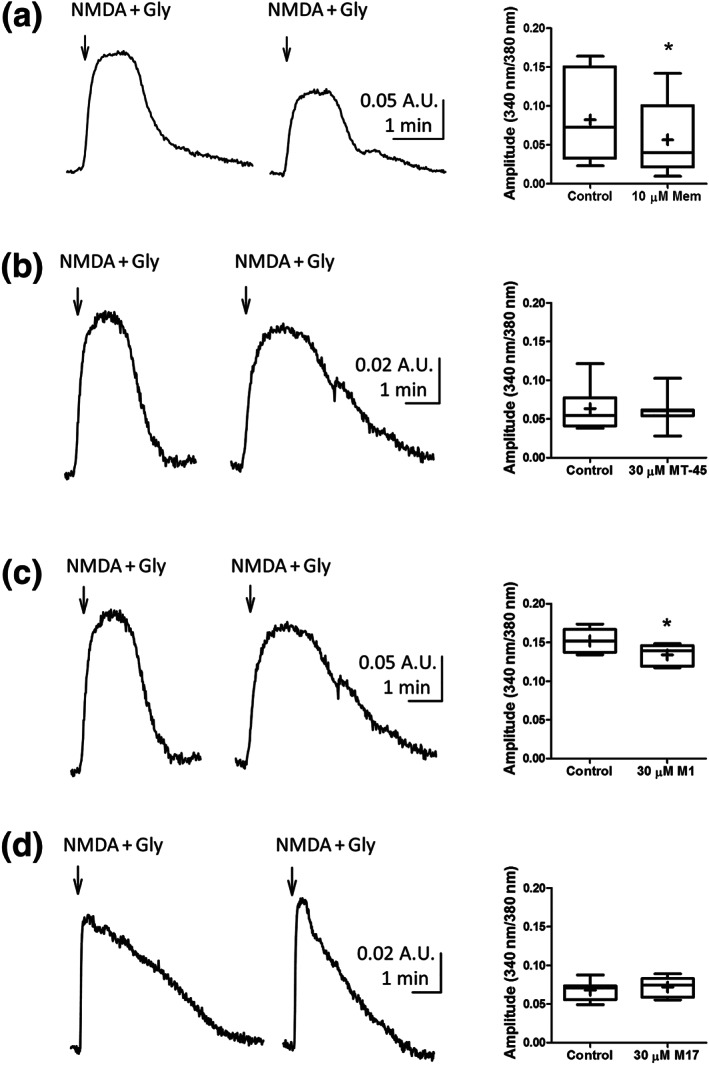
NMDA receptor‐mediated Ca^2+^ responses. Traces are representative examples of intracellular Ca^2+^ transients mediated by the addition of NMDA (30 μM) and glycine (50 μM; indicated by arrow) in the absence (left trace) or presence (right trace) of memantine (10 μM; a), MT‐45 (30 μM; b), M1 (30 μM; c) and M17 (30 μM; d). The amplitudes of the Ca^2+^ transients were calculated, and mean amplitudes are presented as box plots for memantine (a), MT‐45 (b), M1 (c), and M17 (d). A paired *t*‐test was used to compare Ca^2+^ transient amplitudes. There were no statistically significant differences in the Ca^2+^ transient amplitudes caused by MT‐45 or M17. However, both memantine and M1 significantly reduced the Ca^2+^ transient amplitude caused by the addition of NMDA/glycine (*P < 0.05, Student's paired *t*‐test)

M1 shows structural similarities to the dissociative compound, diphenidine. Furthermore, user experiences with MT‐45 posted within online drug forums are consistent with dissociative‐like effects of MT‐45, akin to those reported by users of ketamine (Kjellgren et al., 2016). We therefore tested the hypothesis that MT‐45, M1 and/or M17 inhibit NMDA receptors. We measured intracellular Ca^2+^ levels in Ltk‐ cells stably expressing GluN1 and N2A NMDA receptor subunits. Functional NMDA receptor expression was induced in Ltk‐ cells by overnight incubation in the presence of 1 μM dexamethasone (McIlhinney et al., 1998). Cells were loaded with Fura2‐AM, and intracellular fluorescence was measured (see Section 2). Superfusion of extracellular solution containing 30 μM NMDA and 50 μM glycine induced robust intracellular Ca^2+^ increases, consistent with the response representing activation of NMDA receptors. The NMDA receptor antagonist memantine (10 μM) applied 5 min prior to NMDA and glycine reduced the amplitude of the intracellular Ca^2+^ responses (Figure 6a). We quantified the amplitude of the NMDA and glycine induced intracellular Ca^2+^ response. A paired Student's *t*‐test analysis of mean amplitudes revealed a statistically significant difference in the presence of memantine (Figure 6a box plot).

Using a similar approach, we pre‐applied MT‐45, M1 and M17 (all at 30 μM). None of the drugs tested changed Fura2‐mediated fluorescence immediately upon addition, indicating that they had no direct effects on intracellular Ca^2+^ (data not shown). Figure 6b shows the effects of MT‐45 (30 μM) on NMDA‐ and glycine‐evoked intracellular Ca^2+^ changes. A paired *t*‐test revealed no statistically significant changes in the presence of MT‐45 (Figure 6b box plot). Next we tested the influence of M1 on NMDA receptor‐mediated intracellular Ca^2+^ changes. Pre‐application of M1 (30 μM) caused a small reduction in the NMDA and glycine induced Ca^2+^ response (Figure 6c traces). Comparison of the amplitudes with a paired Student's *t*‐test revealed a statistically significant reduction in the NMDA and glycine induced Ca^2+^ response (Figure 6c box plot). M17, by contrast, did not show the same effects as M1 (Figure 6d), with no statistically significant change to the NMDA receptor‐mediated Ca^2+^ response (Figure 6d).

**FIGURE 7:**
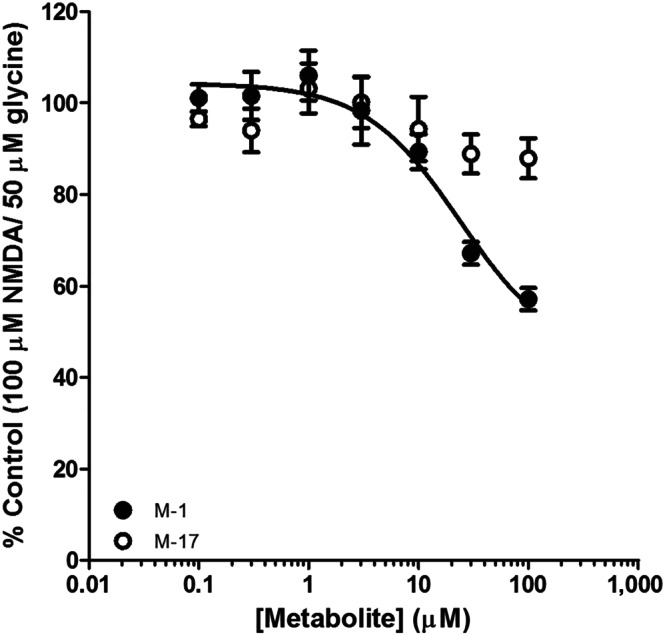
Inhibition of NMDA receptor by MT‐45 metabolites. Concentration–response relationships of M1 (closed circles) and M17 (open circles) mediated inhibition of NMDA (100 μM) and glycine (50 μM) evoked Ca^2+^ responses from Ltk‐ cells. Data are normalised to maximum Ca^2+^ response amplitude in the absence of inhibitors. M17 did not cause inhibition of NMDA receptors. However, there was a concentration‐dependent inhibition of Ca^2+^ responses by M1. The solid line indicates the logistic function fit to the data

We constructed full concentration–response relationships of M1‐ and M17‐mediated inhibition of NMDA receptors. Ltk‐ cells were seeded into 96‐well plates and loaded with Fura2‐AM. Cells were pretreated (for 10 min) with increasing concentrations of M1 and M17 (up to 100 μM). Basal fluorescence values were acquired for 2 min before the addition of NMDA and glycine (at final concentrations of 100 and 50 μM, respectively). Fluorescence ratio changes were monitored for 5 min. Peak values were normalised to controls (without M1 or M17) and plotted as a concentration–response relationship (Figure 7). Consistent with our data in Figure 6, M17 did not show concentration‐dependent inhibition of NMDA receptors. M1, by contrast, inhibited NMDA receptors in a concentration‐dependent manner. We fitted logistics functions to the data and determined the mean (and range) IC_50_ of M1 to be 29 (45) μM (*n* = 7). These data reveal that, while the parent MT‐45 compound does not inhibit NMDA receptors, at least one of its metabolites, M1, may contribute to the dissociative effects reported by users of MT‐45.

### 4 DISCUSSION

This study demonstrates that the apparent potencies of MT‐45 at reducing cAMP levels and recruiting β‐arrestin2 are relatively low when compared to the μ receptor agonists, DAMGO, morphine or fentanyl. However, the addition of a fluorine group at either the second, third or fourth carbon of the first benzine ring increases the apparent potencies of the resulting compound by over 20‐fold. Indeed, the potencies of these fluorinated derivatives are similar to those of morphine and fentanyl. Furthermore, while MT‐45 itself has no effect on NMDA receptor‐mediated elevations of intracellular Ca^2+^, its major metabolite M1, caused a significant inhibition of NMDA‐evoked responses.

Opioid analgesics, including morphine and fentanyl, remain a mainstay for pain relief despite their well‐documented side effects such as opioid‐induced hyperalgesia, respiratory depression, immunosuppression and constipation (Colvin et al., 2019). The use of opioids is further complicated by the development of analgesic tolerance, necessitating progressively higher doses of opioid analgesics. Opioids can also be habit forming, potentially leading to misuse and addiction. Activation of μ receptors and, to a lesser extent, δ‐opioid receptors reduces inhibitory transmission in the ventral tegmental area (Bull, Baptista‐Hon, Lambert, Walwyn, & Hales, 2017). The resulting disinhibition of dopaminergic neurones allows increased dopamine release into the striatum and prefrontal cortex, events implicated in opioid‐induced reinforcement and reward (Fields & Margolis, 2015; Maldonado et al., 1997).

Research in the 1970s seeking the next generation of opioid‐based analgesics generated a number of synthetic opioids, including MT‐45, originally developed as an alternative to morphine (Fujimura et al., 1978; Nakamura & Shimizu, 1976). However, the side effects of MT‐45 were problematic and therefore development was halted. MT‐45 and several other novel synthetic opioids have recently emerged in the U.S. and European recreational drug markets. MT‐45 in particular was implicated in drug‐related deaths in Sweden (Helander et al., 2017), the United States (Papsun, Krywanczyk, Vose, Bundock, & Logan, 2016), and Germany (Fels et al., 2017). MT‐45, its derivatives, and a number of other novel synthetic opioids have sequentially been controlled in the United Kingdom and around the world, since 2014 (Sharma, Hales, Rao, NicDaeid, & McKenzie, 2019). China, widely accepted to be the main production centre for novel synthetic opioids, banned the manufacture and export of MT‐45 from 2017. Legislation to restrict supply, combined with poor user experiences, and reported side effects led to the rapid disappearance of MT‐45 from the illicit market (Helander et al., 2014; Sharma et al., 2019).

The fluorinated derivative of MT‐45, 2F‐MT‐45, discovered in a tablet seized at the 2016 Mantra Festival in Manchester, UK, neither appears on user forums, nor are we aware of its availability from online vendors. Prior to this study, nothing was known about the pharmacology of either the fluorinated derivatives or metabolites of MT‐45. There is a need for more knowledge about the pharmacology of fluorinated derivatives of MT‐45 now that 2F‐MT‐45 has made its way into the recreational drug market.

Activation of μ receptors modulates cellular function and affects receptor recycling through G proteins and β‐arrestin2, respectively (Kang et al., 2015; Koehl et al., 2018). Mounting evidence suggests that β‐arrestin2 recruitment to the μ receptor also engages additional signalling molecules, such as Src kinase, associated with the development of tolerance (Bull, Baptista‐Hon, Sneddon, et al., 2017; Walwyn et al., 2007). User experiences of MT‐45 in online forums recount the development of tolerance (Kjellgren et al., 2016). Other side effects are also mentioned, including nausea and vomiting, constipation, respiratory depression, and loss of motor skills. In addition to tolerance, opioid‐induced respiratory depression and constipation have also been attributed to recruitment of β‐arrestin2 (Bohn et al., 1999; Raehal & Bohn, 2011). However, a recent report suggests that β‐arrestin2 may not participate in opioid‐induced respiratory depression and constipation (Kliewer et al., 2019). It is unclear whether any of the self‐reported users of MT‐45 were also/alternatively consuming its fluorinated derivative.

We compared inhibition of cAMP accumulation (as an assay of G protein signalling) and β‐arrestin2 recruitment by MT‐45, its fluorinated derivatives and metabolites as well as the traditional opioid agonists, DAMGO, morphine and fentanyl. This comparative approach is often used to establish agonist signalling bias. Agonists biased against β‐arrestin2 recruitment and in favour of G protein signalling may exhibit fewer side effects, such as tolerance, respiratory depression and constipation (Madariaga‐Mazón et al., 2017; Manglik et al., 2016).

The potency of MT‐45 as an inhibitor of cAMP accumulation was 10‐fold lower than observed in a recent study of [^35^S]GTPγS binding (Baumann et al., 2018). This may reflect differing μ receptor numbers in our recombinant expression systems and the fact that adenylyl cyclase inhibition lies downstream of G protein activation. Furthermore, the potency of MT‐45 for recruitment of β‐arrestin2 is 20‐fold lower than our cAMP assay. It is important to note that there are differing levels of amplification within the G protein‐mediated cAMP pathway and β‐arrestin2 recruitment. The β‐galactosidase fragment complementation assay, used in this study, measures the recruitment of β‐arrestin2 to μ receptors, requiring molecular proximity of a β‐galactosidase fragment on the μ receptor with its complementary fragment on the β‐arrestin2 molecule, with a stoichiometry of 1:1. Structural models of GPCR–arrestin complexes support the notion that each receptor recruits one arrestin (Kang et al., 2015). Although in some cases arrestins may remain activated after their interaction with a GPRC has terminated, enabling amplification (Latorraca et al., 2018), this is unlikely in the case of the complementation assay, which is irreversible. The lack of amplification is advantageous in this case as it enables the assay to distinguish partial agonists from full agonists. By contrast, the assay of cAMP accumulation occurs at the culmination of several reversible events: first receptor activation, then G protein coupling and finally inhibition of adenylyl cyclase. Furthermore, the stoichiometry of receptors to G proteins will depend on the level of recombinant μ receptor expression. In PathHunter cells μ receptors are over‐expressed, enabling partial agonists to fully inhibit adenylyl cyclase. However, despite these potential limitations, this assay has the advantage of being sensitive, even detecting the activity of weak partial agonists. Consistent with this idea, morphine, fentanyl and MT‐45 and its fluorinated derivatives, partial agonists compared to DAMGO in the β‐arrestin2 recruitment assay, were equally efficacious in the assay of cAMP accumulation. Our observation that 2F‐MT‐45 inhibited β‐arrestin2 recruitment by DAMGO is consistent with the idea that the drug binds to the μ receptor and acts as a partial agonist. Furthermore, M1 and M17, metabolites of MT‐45, which exhibited no detectable stimulation of β‐arrestin2 recruitment to μ receptors, were nevertheless able to act as very weak partial agonists in the cAMP assay.

Their negligible efficacy at the μ receptor implies that M1 and M17 probably do not contribute to any rewarding effects of MT‐45. However, the ability of M1 to inhibit NMDA receptors may contribute to the reported dissociative effects of MT‐45 (Kjellgren et al., 2016). Furthermore, when combined with the μ receptor‐mediated potential for respiratory depression by MT‐45, inhibition of NMDA receptors by M1 may add to the known risk of fatality associated with recreational MT‐45 use (Fels et al., 2017; Helander et al., 2014; Papsun et al., 2016).

The role of fentanyl and its analogues in the current opioid crisis in the United States is well documented (Prekupec et al., 2017; Socías & Wood, 2017). Our findings highlight the potential danger associated with novel fluorinated MT‐45 derivatives, which exhibit higher potency as μ receptor agonists than the parent compound. In this regard, the work reveals the profound influence of minor modifications to the chemical structures of novel synthetic opioids. The recently reported deaths caused by MT‐45 highlights the danger of novel synthetic opioid misuse (Fels et al., 2017; Papsun et al., 2016). The concentration of MT‐45 in one victim's femoral blood was more than sixfold higher than the EC_50_ determined here for μ receptor‐mediated inhibition of cAMP. The substantially higher apparent potencies of fluorinated derivatives of MT‐45 may pose an additional danger to illicit opioid users.

Fluorination of existing psychoactive substances is a common strategy in both medicinal chemistry (Purser et al., 2008; Gillis et al., 2015) and the production of illicit drugs. Such a strategy has been particularly prevalent with synthetic cannabinoid receptor agonists (Banister et al., 2015; Chung et al., 2014). Bioisosteric substitution of a hydrogen atom for fluorine often increases drug potency as has been noted previously for the opioid analgesic viminol, the 2F‐ and trifluoromethyl‐analogues of which are significantly more potent analgesics (Conti, 1979). In addition to increasing potency, addition of fluorine is also likely to increase bioavailability through alteration of physicochemical properties such as pKa. This effect was also noted for the fluorinated analogues of MT‐45 (McKenzie et al., 2018).

Early in 2019, the State Council of the People's Republic of China announced controls for the production and export of fentanyl analogues; although it is too soon to establish the impact, one consequence could be a compensatory increase in the availability of non‐fentanyl novel synthetic opioids on the illicit market. It will be important to rapidly isolate and identify novel synthetic opioids as they emerge and assess their mechanisms of action. This approach, which will require close cooperation between forensic drug researchers and pharmacologists, may provide indications of the potential harm of emerging novel synthetic opioids. In particular, it will be important to alert relevant health care practitioners and the communities exposed to risk, of chemical modifications to existing substances that cause significantly increased potency, such as we observed in the case of fluorinated MT‐45.

## AUTHOR CONTRIBUTIONS

D.T.B.‐H., M.S., and S.S. performed the β‐arrestin recruitment, cAMP and intracellular Ca^2+^ assays. D.T.B.‐H., M.S., S.S. and T.G.H. analysed the data. L.H.A. and C.M. performed chemical syntheses. N.N.D., C.M., D.T.B.‐H., and T.G.H. conceived the study. D.T.B.‐H., C.M., N.N.D. and T.G.H. wrote the paper.

## CONFLICT OF INTEREST

The authors declare no conflicts of interest.

## DECLARATION OF TRANSPARENCY AND SCIENTIFIC RIGOUR

This Declaration acknowledges that this paper adheres to the principles for transparent reporting and scientific rigour of preclinical research as stated in the *BJP* guidelines for Design & Analysis, and as recommended by funding agencies, publishers and other organisations engaged with supporting research.
